# Glatiramer Acetate Increases Phagocytic Activity of Human Monocytes *In Vitro* and in Multiple Sclerosis Patients

**DOI:** 10.1371/journal.pone.0051867

**Published:** 2012-12-20

**Authors:** Refik Pul, Franco Morbiducci, Jelena Škuljec, Thomas Skripuletz, Vikramjeet Singh, Ute Diederichs, Niklas Garde, Elke Verena Voss, Corinna Trebst, Martin Stangel

**Affiliations:** 1 Department of Neurology, Hannover Medical School, Hannover, Germany; 2 Center for Systems Neuroscience, Hannover, Germany; Klinikum rechts der Isar der Technischen Universitaet Muenchen, Germany

## Abstract

Beside its effects on T cells, a direct influence on cells of the myelo-monocytic lineage by GA becomes evident. Recently, we demonstrated that GA drives microglia to adopt properties of type II antigen presenting cells (APC) and increases their phagocytic activity. In the present work, we focused on human blood monocytes in order to examine whether GA may increase phagocytic activity *in vivo* and to evaluate the molecular mechanisms explaining this new discovered mode of action.

Peripheral blood mononuclear cells (PBMC) were obtained using a Biocoll-Isopaque gradient and monocytes were subsequently isolated by using CD14 MicroBeads. Phagocytic activity was determined by flow cytometric measurement of the ingestion of fluorescent beads. Flow cytometry was also used to assess monocytic differentiation and expression of phagocytic receptors. Monocytes of GA treated MS patients exhibited a significantly higher phagocytic activity than those of healthy controls or non-treated MS patients. *In vitro*, a significant phagocytic response was already detectable after 1 h of GA treatment at the concentrations of 62.5 and 125 µg/ml. A significant increase at all concentrations of GA was observed after 3 h and 24 h, respectively. Only monocytes co-expressing CD16, particularly CD14^++^CD16^+^ cells, were observed to phagocytose. Treatment of monocytes with IL-10 and supernatants from GA-treated monocytes did not alter phagocytosis. We observed a decrease in CD11c expression by GA while no changes were found in the expression of CD11b, CD36, CD51/61, CD91, TIM-3, and CD206. In our blocking assays, treatment with anti-CD14, anti-CD16, anti-TIM3, anti-CD210, and particularly anti-CD36 antibodies led to a decrease in phagocytosis.

Our results demonstrate a new mechanism of action of GA treatment that augments phagocytic activity of human monocytes *in vivo* and *in vitro*. This activity seems to arise from the CD14^++^CD16^+^ monocyte subset.

## Introduction

Circulating blood monocytes are critical effectors of the innate immune response regulating the adaptive immunity [1]. During inflammatory disorders, such as multiple sclerosis (MS), monocytes are repeatedly recruited from the periphery, thereby reinforcing the local inflammatory reaction within the central nervous system (CNS) [2], [3]. Inflammatory lesions in the CNS of MS patients and animals with experimental autoimmune encephalomyelitis reveal an abundant presence of mononuclear phagocytic macrophages which originate from resident microglia and infiltrating monocytes [4], [5]. Equipped with a large array of scavenger receptors these cells are involved in repair mechanisms by removing myelin breakdown products since it has been identified that myelin debris is a substantial inhibitor of remyelination [6]. Depletion of blood monocytes resulted in impaired remyelination underlining that this replenishing pool of myeloid cells is required to meet the demands for efficient repair [7], [8].

Among the approved drugs for MS treatment, GA has been shown to exert immunomodulatory effects not only on T cells but also on cells of the myelo-monocytic lineage [9], [10], [11], [12], [13], [14]. Accordingly, treatment of dendritic cells (DCs) with GA increased the secretion of the anti-inflammatory cytokine interleukin (IL)-10 and decreased that of the major Th1 polarizing factor IL-12 as well as tumor necrosis factor (TNF)-α [9], [11]. Moreover, GA broadly inhibited the activation of monocytes and promoted development of type II monocytes which secreted more IL-10 and transforming growth factor-β, and less IL-12 and TNF-α [14]. Independently of antigen specificity, these monocytes directed differentiation of Th2 cells and T regulatory cells (Tregs) [15]. Adoptive transfer of type II monocytes reversed EAE, suppressed Th17 cell development, and promoted both Th2 differentiation and expansion of Tregs in recipient mice [10], [15].

Recently, we demonstrated that GA drives microglia to adopt a type II APC similar to monocytes and DCs suggesting a general effect on myelo-monocytic cells [16]. We additionally discovered that GA directly and distinctly increases phagocytic activity of primary rat microglia. In the present study, we focused on human blood monocytes and examined whether this effect can be observed in monocytes of MS patients treated with GA. Since GA has been shown to bind strongly to the integrin macrophage-1 antigen (CD11b/CD18) and to increase the level of the T cell immunoglobulin mucin-3 (TIM-3) mRNA in peripheral blood mononuclear cells (PBMC), we performed further *in vitro* experiments to evaluate possible mechanisms explaining the increased phagocytic activity [17], [18].

## Materials and Methods

### Patients

A total of 13 GA treated (20 mg per day subcutaneously) and 20 non-treated subjects who met the criteria for relapsing-remitting MS according to revised McDonald criteria (2005) were enrolled [19]. Age of patients ranged from 24 to 61 years with Expanded Disability Status Scale (EDSS) from 1.5 to 5.5 (Table 1). The control group consisted of 10 healthy volunteers.

**Table 1:** Patient characteristics (n.a. = not applicable).

Proband group	N (f/m)	Age, years	Duration of disease, years	Duration of GA treatment, years	EDSS
GA treated	13 (13/0)	48 (37–56)	11 (2–22)	4 (1–10)	2 (1–5.5)
non-treated	20 (17/3)	37 (24–61)	4 (1–26)	n.a.	1.5 (0–3.5)
healthy controls	10 (6/4)	35 (24–58)	n.a.	n.a.	n.a.

In the GA treated group 4 probands were taking additional drugs due to arterial hypertension (nebivolol 5 mg once daily), hypercholesterolemia (simvastatin 20 mg once daily), spasticity (baclofen 30 mg three times daily), mood alteration (sertralin 50 mg once daily, carbamazepin 600 mg twice daily) and hypothyreosis (L-thyroxin 75 µg or 125 µg once daily). In each of the non-treated and healthy control group one subject was taking concomitant medication because of arterial hypertension (lisinopril 10 mg and hydrochlorothiazide 12.5 mg once daily; carvedilol 12.5 mg and olmesartan 10 mg once daily). None of the probands had received any immunosuppressive or differing immunomodulating treatment for at least six month prior to enrolment. None of them had any infections at the time of presentation. All donors provided written consent, with the approval of the Institutional Review Board of the Hannover Medical School, for the collection of peripheral blood and subsequent analysis.

### Cell isolation

#### Peripheral blood mononuclear cells

Blood was obtained by venous puncture and drawn in ethylene diamine tetraacetic acid tubes. PBMC were separated from whole blood samples on a continuous Biocoll (Biochrom, Berlin, Germany) density gradient (1.077 g/ml). After centrifugation (300×*g*, 25 min) cells at the interface were collected, washed three times with phosphate buffered saline without Ca^2+^ or Mg^2+^ (PBS; Biochrom).

#### Monocytes

Monocytes were isolated from PBMC using CD14 MicroBeads™ (Miltenyi Biotec, Bergisch Gladbach, Germany) and passing through a MACS column (autoMACS™ Separators, Miltenyi Biotec) to positively select for CD14^+^ cells by immunomagnetic selection, according to the manufacturer's instructions. This procedure yielded at least a 90% pure population of monocytes, as assessed by fluorescence-activated cell sorter analysis.

#### Cell culture

Isolated CD14^+^ monocytes were resuspended in Dulbecco's Modified Eagle Medium (DMEM; Gibco, Karlsruhe, Germany) supplemented with 10% fetal calf serum (Biochrom) and penicillin plus streptomycin (Gibco). They were plated at 3×10^5^ cells per well in 6-well plates (Nunclon™Surface; Nunc, Roskilde Site, Denmark) in 2 ml medium and allowed to adhere for 30 minutes at 37°C in humidified air containing 5% CO_2_, and nonadherent contaminants were removed with two washes with PBS (Biochrom). The adherent monocytes were cultured in DMEM with the aforementioned supplements (medium) and cultured over night before experimentation. The medium was then replaced with fresh medium containing GA (TEVA Pharma GmbH, Kirchzarten, Germany), mannitol (the vehicle control contained in GA; Merck, Eurolab GmbH, Germany), human serum albumin (HA; Baxter GmbH, Unterschleißheim, Germany) or cytochalasin D (Sigma-Aldrich, St. Louis, USA) as indicated. After different incubation periods cells were removed and used for further experimentation.

### Measurement of cell viability and apoptosis

After 24h-treatment of monocytes treated with GA or mannitol, supernatants were removed and cell viability was assessed by resazurin (AlamarBlue®; Biosource, Solingen, Germany) diluted 1∶10 with medium for 3 h at 37°C. Optical densities were measured at 620 nm emission using a spectrophotometer (Tecan Sunrise, Crailsheim, Germany). Apoptosis was determined using annexin V and propidium iodide (PI) dual staining kit (BD Biosciences Pharmingen, San Diego, CA, USA) following the manifacturer's protocol using a FACScalibur™ flow cytometer (Becton-Dickinson, San Jose, CA, USA). Data were analyzed with CellQuest™ software (Becton-Dickinson). In both assays duplicate measurements were averaged from three independent experiments.

### Immunocytochemistry and confocal microscopy

6×10^4^ monocytes were plated onto 12 mm glass cover slips, placed in 4-well plates (Nunc), and treated for 24 h with 31.25 µg/ml GA, HA, or 61.5 µg/ml mannitol. After 30 minutes incubation with fluorescent latex beads (Fluoresbrite™ YG carboxylate microspheres; 1 µm diameter; Polysciences, Eppelheim, Germany) at 37°C, cover slips were washed three times and cells were stained with the CD14 antibody (primary: rabbit anti-human IgG, Abcam, Cambridge, UK; secondary: goat anti-rabbit IgG, AbD Serotec, Düsseldorf, Germany) at 1∶100 dilution in DMEM+. They were fixed with 4% paraformaldehyde (Sigma-Aldrich, Steinheim, Germany) and visualized with Alexa-fluor 555 (at 1∶300 dilution in PBS, goat anti-rabbit IgG_2a_, Invitrogen, California, USA). After mounting with Mowiol (Calbiochem, San Diego, CA, USA) containing 4′,6-diamidino-2-phenylindole (Invitrogen, Carlsbad, CA, USA) cells were photographed (Olympus BX61 with camera DP72, Olympus, Tokyo, Japan). Confocal scans were performed using an inverted Carl-Zeiss LSM510 with an Axiovert 200 M microscop and a Zeiss Plan Apo 60×, NA 1.4 oil immersion objective (Carl-Zeiss, Jena, Germany). Pictures were analyzed using the LSM software package version 3.5 (Carl Zeiss MicroImaging, Jena, Germany).

### Flow cytometrical analysis

#### Phagocytosis assay

Monocytes were immediately isolated after each venipuncture and 1 µm-diameter fluorescent latex beads (Polysciences) were added in a final dilution of 1∶200 into a FACS tube (BD Falcon Round-Bottom Tube; BD Biosciences) containing 1×10^6^ (patient blood) or 3×10^5^ cells (all further *in vitro* experiments), respectively. After 30 minutes of incubation at 37°C monocytes were washed with PBS and centrifuged (10 min, 240×*g*). This washing step was repeated three times. Polystyrene beads (Polysciences) were added to wells containing 3×10^5^ cells. After two washing steps, cells were detached by vigorous pipetting and washed again three times with PBS. Cells were then resuspended in FACS-flow (Becton-Dickinson) and examined on a FACScalibur Becton-Dickinson flow cytometer. Forward scatter, side scatter, and green fluorescence channel (FL1) were used to quantify phagocytosis. Unbound beads exhibiting a low forward scatter and a high fluorescence signal were excluded from the analysis by gating.

The mean fluorescence intensities (MFI) were calculated by subtracting the background MFI (sample without beads). In all *in vitro* assays, the MFI of each substance was subtracted by the MFI of this substance without beads. The resulting MFI is considered to be an equivalent of the total number of latex beads phagocytosed by a given number of cells [20].

#### Coating of beads

Native lyophilized and plasminogen-depleted fibrinogen from human plasma (Calbiochem, Darmstadt, Germany) was adjusted at a concentration of 5 mg/ml using PBS. Beads were diluted by 1∶10 in an appropriate volume of fibrinogen. The bead-fibrinogen solution was then constantly mixed and incubated for 4 h at 37°C. After a subsequent centrifugation step (1200 g, 15 minutes), the supernatant was removed and the beads were resuspended in PBS. For the phagocytosis assays the fibrinogen-coated beads were added in a final dilution of 1∶200 into a FACS tube.

#### Endocytosis of human Ox-LDL

3×10^5^ PBMC were first treated with GA at the indicated concentrations for 3 h and then cultured in the presence of 10 µg/ml human DiO(3′3,-dioctadecyloxacarbocyanine)-oxidized low density lipoprotein (DiO-OxLDL, Kalen Biomedical, Montgomery Village, USA) for 1 to 12 h at 37°C in humidified air containing 5% CO_2_. The anti-CD36 antibodies (clone 255606 and FA6–152) were added to the cultures 9 h before adding GA at a concentration of 1 µg/ml. After incubation, cell plates were put on ice and cells were detached by vigorous pipetting. Cells were then centrifuged, washed, and resuspended in PBS containing 1% fetal calf serum and 0.1% sodium azide. The ingestion of DiO-OxLDL was immediately evaluated in a flow cytometer using the green fluorescence channel. For quantifying the percentage of “negative” monocytes, i.e. cells that did not ingested DiO-OxLDL, corresponding samples without DiO-OxLDL served as control. Monocytes were gated within the PBMC according to their light scatter characteristics and 10.000 events were recorded. Data were analyzed using the software FCS4 Express™ (De Novo Software, Los Angeles, USA).

#### Antibodies

PE-conjugated anti-CD11b IgG_2b_ (Cat. No. FAB16991A, Clone 238446; R&D Wiesbaden-Nordenstadt, Germany), anti-CD11b IgG_1_κ (Cat. No. 301312, Clone ICRF44; Biolegend, San Diego, CA, USA), APC-conjugated anti-CD11c IgG_1κ_ (Cat. No. 559877, Clone B-Ly6; BD Biosciences), PE-conjugated anti-CD14 IgG_1_ (Cat. No. FAB3832, Clone 134620; R&D, Wiesbaden-Nordenstadt, Germany), PE-conjugated anti-FcγRIIIa/b IgG_2a_ (Cat. No. FAB2546P, Clone 245536; R&D), anti-CD18 IgG_1κ_ (Cat. No. 302112, Clone TS1/18, Biolegend), PE-conjugated anti-CD36 IgG_2b_ (Cat. No. FAB19551P, Clone 255606; R&D), anti-CD36 IgG1 (Cat. No. 01436, Clone FA6–152, StemCell Technologies, Köln, Germany), PE-conjugated anti-CD51/61 IgG_1_ (Cat. No. FAB3050P, Clone 23C6; R&D), FITC-conjugated anti-CD91/LRP1 IgG_1_ (Cat. No. SM1729F; Acris, Herford, Germany), anti-CD206 IgG_1κ_ (Cat. No. 321111, Clone 15-2, Biolegend), anti-CD210 IgG_2aκ_ (Cat. No. 308806, Clone 3F9, Biolegend), PE-conjugated anti-TIM-3 IgG_2a_ (Cat. No. FAB2365, Clone 344823; R&D), mouse IgG_1κ_ (Cat. No. 400123, Clone MOPC21, Biolegend), mouse IgG_1κ_ (APC, Cat. No. 17-4714, Clone P3.6.2.8.1, eBioscience, Frankfurt, Germany), rat IgG_1κ_ (APC, Clone eBRG1, eBioscience), mouse IgG_1κ_ (PE, Cat. No. 550616, Clone MOPC-31C, BD Bioscience), mouse IgG_1_ (FITC, Cat. No. IC002F, Clone 11711; R&D), mouse IgG_2aκ_ (PE, Cat. No. 553457, Clone G155–178; BD Biosciences), rat IgG_2a_ (Cat. No. 400515, Clone RTK2758, Biolegend), rat IgG_2b_ (PE, Cat. No. 12-4031, eBioscience), mouse IgG_2b_ (PE, Clone 20116.11).

#### Expression of surface proteins

3×10^5^ Monocytes were incubated with conjugated antibodies or isotype controls for 30 min at 4°C as indicated, according to the protocol of each manufacturer. After washing twice with PBS, cells were analyzed by flow cytometry (FACScan, BD Biosciences) using Cell Quest software (BD Biosciences). For each analysis, at least 10000 events were collected and specific proteins in each sample were quantified by the mean fluorescence intensity.

#### Blocking assays

After an antibody pre-treatment of 3×10^5^ PBMC for 8 h, 31.25 µg/ml GA was added to the wells as indicated and cells were additionally incubated at 37°C in humidified air containing 5% CO_2_ for 3 h. Phagocytosis of polystyrene beads was quantified as mentioned above.

### Statistical analysis

SigmaPlot® software V11.0 was used for statistical analysis. For each assay, normality of data distribution was tested with the Shapiro-Wilk test. Statistical analysis was performed using one-way analysis of variance (ANOVA) followed by the Bonferroni post-hoc test. For each assay a p value <0.05 was considered as statistically significant. Significant effects are indicated by asterisks (*p<0.05; **p<0.01; ***p<0.001).

## Results

### GA treatment increases phagocytic capacity of human monocytes *in vivo*

Of the 33 MS patients enrolled, 13 were injecting GA, and 20 were without any treatment. The proportion of female in both groups was considerably high (100% and 85%, respectively) whereas the female rate in the healthy control group was about 60% (Table 1). Patients on GA were on average 11–13 years older than subjects of the non-treated and healthy controls. Their mean duration of disease was 7 years longer than the non-treated group (Table 1). Despite this imbalance, there was just a small difference of 0.5 in the mean EDSS between both groups (Table 1). With respect to our previous report on increased microglial phagocytosis by GA, we sought to examine whether GA therapy alters monocytic phagocytosis *in vivo* [16]. Accordingly, CD14^+^ monocytes were isolated from each donor yielding with a 90% purity with less T (CD3^+^) or B cell (CD19^+^) contamination. Phagocytosis was determined by the cells' ability to engulf 1 µm-diameter Fluoresbrite® YG Carboxylate Microspheres without any preceding cell stimulation.

**Figure 1:**
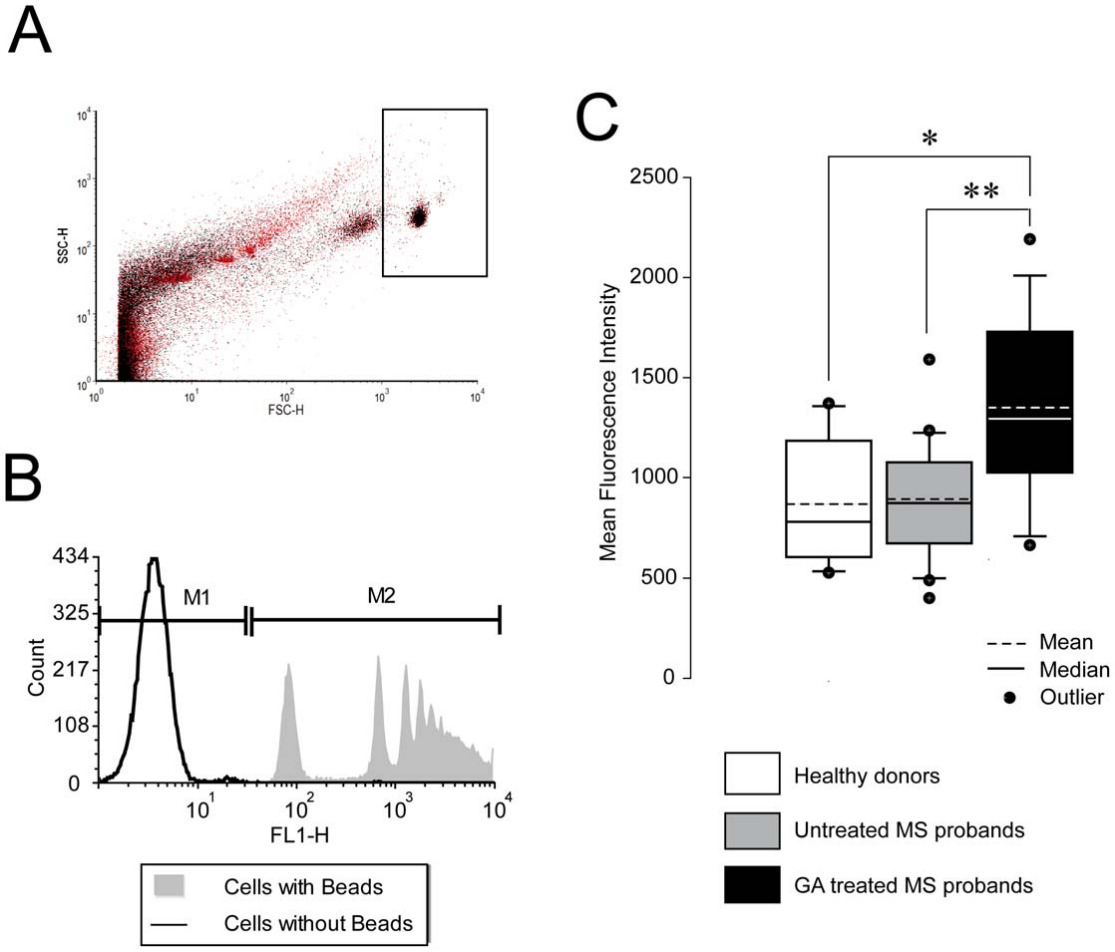
*Ex vivo* assessment of phagocytosis. **A.** After MACS separation monocytes were gated according to their properties in the forward and side scatter. **B.** Phagocytosis of non-opsonized fluorescent polystyrene-based latex beads. Each mean fluorescence is considered to be an equivalent of the total number of latex beads phagocytosed by 1×10^6^ cells. All mean fluorescences are related to the background control (cells without beads). **C.** Monocytes from glatiramer acetate (GA) treated MS patients (n = 13) exhibit a significantly higher phagocytic activity than those of healthy donors (n = 10) or non-treated MS patients (n = 20). Significant effects are indicated by asterisks (*p<0.05 and **p<0.01 using Bonferroni's Multiple Comparison Test) as determined by one-way ANOVA.

Monocytes from the circulation of GA treated subjects exhibited a significantly higher phagocytic activity (MFI 1351.9±440.0 Standard deviation (SD)) as compared to healthy (p = 0.005) or non-treated MS patients (p = 0.001) (Fig. 1C). No statistical difference (p = 0.103) was observed between healthy donors (MFI 870.6±297.2 SD) and non-treated MS patients (MFI 884.4±285.7 SD) (Fig. 1). Moreover, the phagocytosis assay was performed at a fixed monocyte count of 10^6^ cells with a subsequent flow cytometric analysis of 10000 events.

### High concentrations of GA and mannitol are cytotoxic to monocytes *in vitro*

Prior to the *in vitro* experiments, cytotoxicity of GA was assessed to obtain a concentration range in which effects that can be related to cell viability are excluded. Accordingly, resazurin, a stable, water-soluble and non-toxic dye, was used for quantifying the *in vitro* viability of monocytes obtained from healthy donors. Viable cells continuously convert the oxidized form of resazurin to the reduced form by mitochondrial enzyme activity. This metabolic activity was not impaired by GA at a concentration range of 15.625 and 125 µg/ml but a significant decrease was observed at a concentration of 250 µg/ml (p = 0,025) declining further at 500 µg/ml (p<0,001) (Table 2). The amount of oxidized resazurin was 87%±4.11 SD (250 µg/ml GA) and 76.12%±10.65 SD (500 µg/ml GA), respectively, of the medium control. This could only be partially attributed to mannitol that only slightly reduced the amount of oxidized resazurin at 1000 µg/ml (83%±3.25 SD; p = 0.011) which is contained in 500 µg/ml GA.

**Table 2:** Evaluation of cell viability by the alamar blue® (resazurin) dye assay after 24 h of treatment with glatiramer acetate (GA) or the mannitol vehicle control.

Treatment	Percentage of viable cells	Treatment	Percentage of viable cells
Medium	100% (±0)		
GA 15.625 µg/ml	98.07% (±1.92)	Mannitol 31.25 µg/ml	97.24 (±1.98)
GA 31.25 µg/ml	98.67% (±1.87)	Mannitol 62.5 µg/ml	98.42 (±1.85)
GA 62.5 µg/ml	97.78% (±1.94)	Mannitol 125 µg/ml	95.23 (±0.96)
GA 125 µg/ml	95.23% (±2.89)	Mannitol 250 µg/ml	89.08 (±0.42)
GA 250 µg/ml	87.44% (±4.11)^*^	Mannitol 500 µg/ml	89.33 (±1.22)
GA 500 µg/ml	76.12 (±10.65)^***^	Mannitol 1000 µg/ml	82.96 (±3.25)^*^

Optical densities (ODs) were normalized to those of untreated control and expressed in percentages as mean ± SEM of three independent experiments. A decrease in cell viability was observed at higher concentrations of GA (250–500 µg/ml) and to a lesser extent for the mannitol vehicle control. Significant effects vs. medium control are indicated by asterisks (*p<0.05, **p<0.01, and ***p<0.001 using Bonferroni's Multiple Comparison Test) as determined by one-way ANOVA.

Since it has been reported that GA induces pro-apoptotic mechanisms in peripheral lymphocytes by involving Bcl-2, Bax, and Cyt-c, apoptosis was excluded by staining with FITC annexin V in conjunction with the vital dye PI within the concentration range of 15.625 and 125 µg/ml GA [21] (Table 3). All further measurements were limited to concentrations within this range.

**Table 3:** Average mean (± standard deviation) of the percentages of monocytes positive for either annexin V, PI or both obtained from gate properties of the FL1 and FL3 sensor.

Treatment	Annexin V (positive)	Annexin V & PI (positive)	PI (positive)
Medium	31.12% (±5.50)	19.52% (±12.5)	3.49% (±4.54)
GA 15.625 µg/ml	23.84% (±16.3)	20.35% (±18.0)	4.87% (±9.74)
GA 31.25 µg/ml	26.78% (±12.8)	19.03% (±11.55)	4.63% (±0.96)
GA 62.5 µg/ml	26.78% (±15.0)	21.74% (±15.15)	4.42% (±0.70)
GA 125 µg/ml	27.12% (±16.9)	16.45% (±6.92)	4.10% (±1.06)

### GA increases phagocytosis *in vitro*

Monocytes from healthy donors were isolated by MACS separation from PBMC and gated as shown in Fig. 1. The MACS isolated monocytes were used to assess the effects of the dose and time course of GA on the ingestion of non-opsonized polystyrene-based latex particles. These particles offer a simple, rapid, and highly sensitive way to determine phagocytosis *in vitro*. We decided to use 1 µm-diameter of those polystyrene beads to attain a maximum rate of phagocytosis [22].

**Figure 2:**
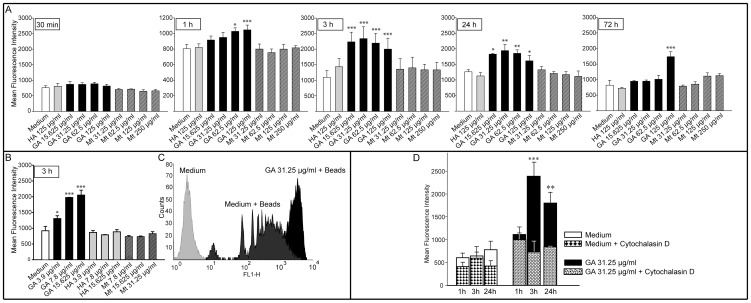
*In vitro* assessment of phagocytosis. **A–B, D.** Data are expressed as means of the mean fluorescence intensities (MFI) ± SEM of three independent experiments. **A–D.** The MFI displays the amount of incorporated fluorescent latex particles phagocytosed by 3×10^5^ cells. Significant effects vs. medium control(s) are indicated by asterisks (*p<0.05, **p<0.01, and ***p<0.001 using Bonferroni's Multiple Comparison Test) as determined by one-way ANOVA. **A.** Monocytes isolated from healthy donors were used to assess the dose and time course of glatiramer acetate (GA) on the ingestion of non-opsonized polystyrene-based latex particles. **B.** After 3 h of incubation low concentrations of GA significantly increased phagocytosis. **C.** Histogram shows the distinct shift of the MFI after 3 h of GA treatment. One representative experiment is shown. **D.** Adhesion of polystyrene beads did not have a relevant impact on the MFI as shown by cytochalasin D (50 µM) experiments.

Intriguingly, a significant increase in phagocytosis was already detectable after 1 h of GA treatment at concentrations of 62.5 µg/ml (MFI 1026.8±77.5; p = 0.011) and 125 µg/ml (MFI 1045.9±SD 107.5; p = 0.005) as compared to the untreated medium control (Fig. 2A). After 3 h, a significant increase at all concentrations of GA tested was observed (Fig. 2A). After 24 h, a slight decrease in bead incorporation was observed at all GA concentrations (Fig. 2A). After a longer incubation period of 72 h, phagocytosis remained significantly increased only at the highest GA concentration (MFI 1737.1±SD 277.2; p<0.001) (Fig. 2A). Neither the unspecific protein control (125 µg/ml HA) nor the mannitol vehicle control at pertinent concentrations significantly altered bead engulfment at any of these conditions (Fig. 2A).

**Figure 3:**
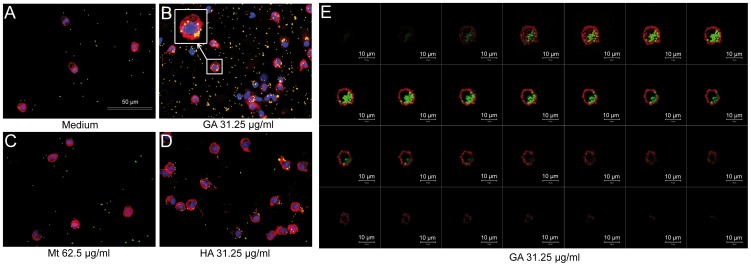
Visualization of phagocytosis. **A–D.** Immunocytochemistry showed distinct phagocytosis of polystyrene beads (green) by monocytes treated with 31.25 µg/ml glatiramer acetate (GA) for 24 h (red, labelled with anti-CD14 antibody) as compared to medium, human serum albumin (HA), and mannitol (Mt) vehicle control. Scale bar: 50 µm. **E.** Representative z-stack series of confocal microscopic images demonstrate that the particles were completely internalised and not merely attached to the outer membrane. Scale bar: 10 µm.

According to our results, phagocytosis peaks after 3 h of GA treatment and diminishes within 2–3 days depending on the concentration used. At this point in time, we also observed significant effects for low concentrations like 3.9 µg/ml (MFI 1307.6±SD 154.2; p = 0.013) and 7.8 µg/ml of GA (1974.6±SD 13.1; p<0.001) (Fig. 2B). The immunocytochemistry revealed that some of the GA treated monocytes indeed take up more beads as compared to the controls (medium, human albumin, or mannitol) (Fig. 3A–D). However, it has also to be mentioned that there are as well monocytes which did not ingest any bead suggesting that not all monocytes are involved in bead uptake.

Cytochalasin D, a substance known to inhibit phagocytosis but not the adsorption of beads, was then used to differentiate surface-attached from ingested particles (Fig. 2D). Cells in medium, cells treated with 50 µM cytochalasin D, and the combination of GA plus cytochalasin D did not differ statistically (p = 0.262) (Fig. 2D). Confocal microscopy was additionally used to ensure that the particles were internalised and not merely attached to the outer membrane of monocytes (Fig. 3E).

### Higher phagocytic activity of CD14^++^CD16^+^ cells

**Figure 4:**
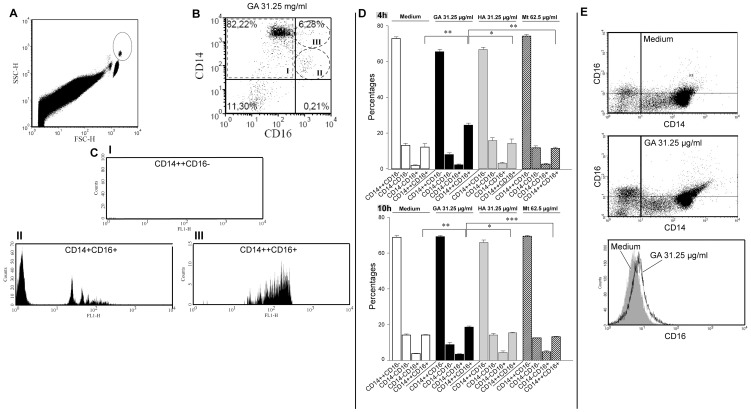
Analysis of monocyte subsets. **A–B.** Gated monocytes in a forward vs. side scatter dot-plot analysis of peripheral blood mononuclear cells. Monocytes were then gated according to their surface expression of CD14 and CD16. Flow cytometric analysis of phagocytosis revealed that CD14^++^CD16^+^ monocytes (gate III) engulfed polystyrene beads more effectively than the other subsets (gate I and II). **CI–III.** The mean fluorescence intensities represent the amount of incorporated fluorescent latex particles phagocytosed by 3×10^5^ cells. **D.** Increase in the percentage of CD14^+^CD16^+^ monocytes after 4 h and 8 h of treatment with 31.25 µg/ml glatiramer acetate (GA) in MACS isolated monocytes. Data are expressed as mean percentages ± SEM of three independent experiments. Significant effects vs. controls are indicated by asterisks (*p<0.05, **p<0.01, and ***p<0.001 using Bonferroni's Multiple Comparison Test) as determined by one-way ANOVA. **E.** Slight but not significant increase of CD16 expression after GA treatment.

Blood monocytes represent a heterogeneous cell population, which can be subdivided into at least three populations, i.e. the CD14^+^CD16^−^ classical monocytes, the CD14^+^CD16^+^ non-classical monocytes, and the CD14^++^CD16^+^ intermediate monocytes [23]. Almost all blood monocytes express the lipopolysaccharide (LPS) receptor antigen CD14, which differs in its cell surface density. The major fraction of monocytes is strongly positive for this antigen and only few cells show co-expression with the Fcγ receptor III (FcγRIII), the CD16 antigen (Fig. 4B) [24].

After treating PBMC with GA for 3 h, monocytes within the PBMC were gated according to their characteristic light scatter pattern and then again gated for CD14 and/or CD16. Interestingly, our results reveal that only cells, which are co-expressing CD16, engulfed polystyrene beads whereas monocytes lacking CD16 did not phagocytose (Fig. 4A). Moreover, amongst CD16 positive cells higher expression of CD14 correlated with an increased phagocytic capacity (CD14^+^CD16^+^: mean MFI 54.3±SD 22.5; CD14^++^CD16^+^: mean MFI 144.2±SD 15.1; Student t-Test p = 0.005).

### GA induces a shift towards CD14^++^CD16^+^ monocytes

Cell surface staining after an incubation period of 4 and 10 h revealed that treatment with GA increased the proportion of CD14^++^CD16^+^ monocytes (4 h: mean percentage 24.4±SD 2.1; 10 h: mean percentage 18.6±SD 1.1) as compared to all controls used (Fig. 4D). This increase was more distinct after 4 h than after 10 h suggesting that after longer incubation the proportion of CD14^++^CD16^+^ cells decline back to percentages similar to those of controls.

Within the CD14-positive cells the percentages of CD14^++^CD16^−^/CD14^++^CD16^+^ were as follows: Medium 4 h: 85.75%/14.27%, 10 h: 82.92%/17.08%; 31.25 µg/ml GA 4 h 72.86%/27.14%, 10 h: 78.83%/21.17%; 31.25 µg/ml HA 4 h: 82.36%/17.64%, 10 h: 81.1%/18.92%; 62.5 µg/ml Mt 4 h: 86.68%/13.32, 10 h: 84.02%/15.98%. These results indicate that GA treatment induces a phenotypic shift from CD14^++^CD16^−^ towards CD14^++^CD16^+^ cells.

Flow cytometric analysis of monocytes showed a slight increase in the expression of CD16 after 4 h of GA treatment which was not significant as compared to control (p = 0.059) (Fig. 4E).

### GA mediated rapid phagocytosis is not induced by a soluble monocytic factor

**Figure 5:**
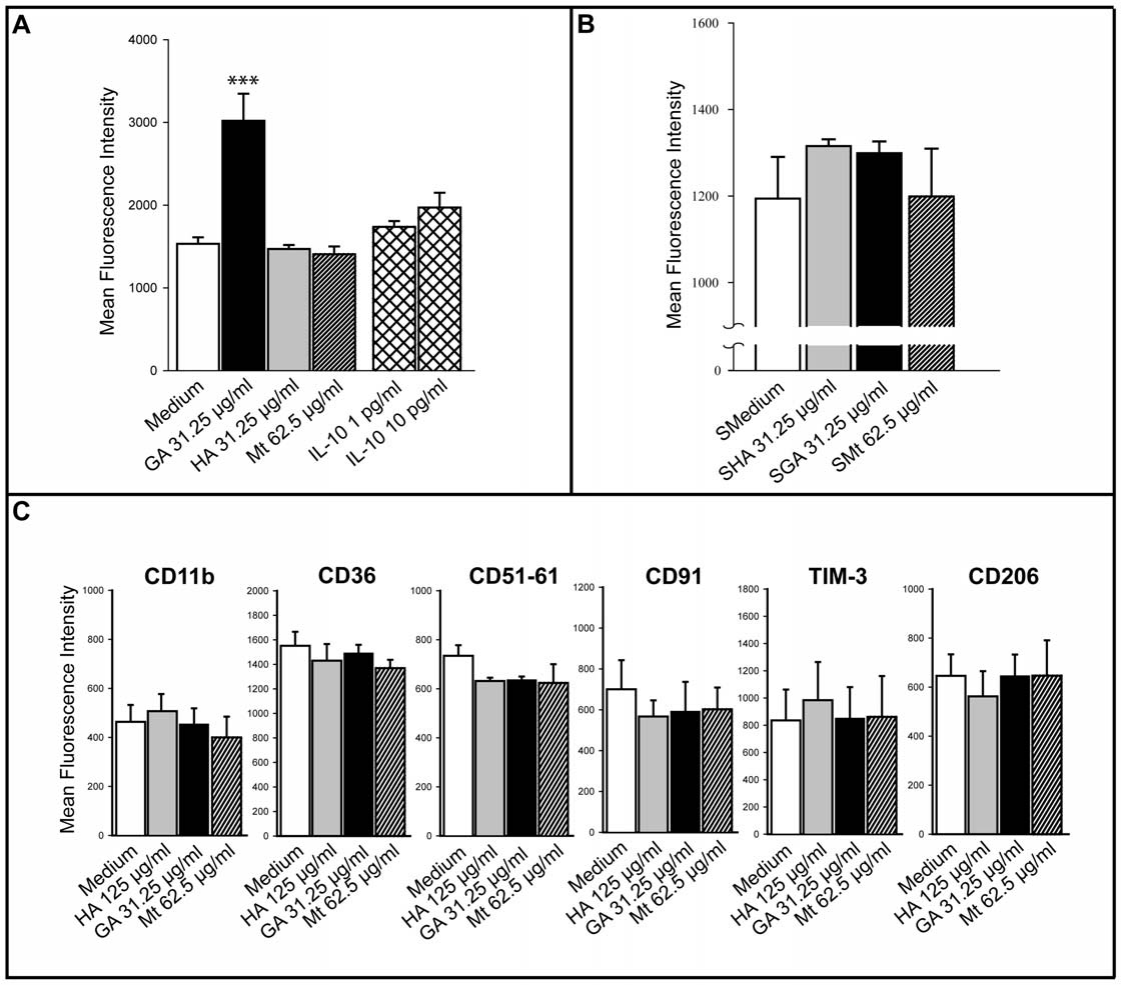
Exclusion of soluble factors led to the analysis of the expression of several phagocytic receptors. **A–B.** The amount of incorporated fluorescent latex particles phagocytosed by 3×10^5^ MACS isolated monocytes is displayed by the mean fluorescence intensities. Data are expressed as means of the mean fluorescence intensities (MFI) ± SEM of three independent experiments. A significant effect vs. medium control is indicated by asterisks (*p<0.05 and ***p<0.001 using Bonferroni's Multiple Comparison Test) as determined by one-way ANOVA. **A.** Monocytes were treated for 3 h with the denoted substances. In particular, IL-10 treatment did not increase phagocytosis. **B.** Treatment of freshly isolated monocytes for 6 h with conditioned supernatants from monocytes, which were previously treated for 12 h with GA, did not have an impact on phagocytosis. **C.** Surface expression of several receptors or subunits of such receptors involved in phagocytosis after a treatment period of 24 h.

In our previous work, we demonstrated that GA increases IL-10 secretion in a dose-dependent fashion. Treatment of 4×10^5^ microglia with 125 µg/ml GA led to a mean secretion of 158.8 pg/ml IL-10 [16]. This cytokine is known to activate scavenger receptors up-regulating monocytic phagocytosis [25], [26], [27]. Thus, it stood to reason that the observed phagocytic effect may be due to an autocrine loop of IL-10. However, treatment of monocytes with 1 and 10 µg/ml of IL-10 for 3 h did not increase phagocytosis (Fig. 5A).

To examine whether increased phagocytosis is due to another soluble factor, monocytes were first stimulated by GA or respective controls. After 12 h the supernatants of these monocytes were completely discarded in order to remove the factors contained in these supernatants. Thereafter, fresh culture medium was added to these “pre-stimulated cells”. After an additional 12 h of incubation, the supernatants were transferred to freshly isolated, non-treated, monocytes, which then were incubated for 6 h whereupon the phagocytosis assay was performed. Unexpectedly, the supernatants from GA-treated monocytes did not show any effect on phagocytosis ruling out the possibility that a soluble factor might have mediated the increase of phagocytosis (Fig. 5B).

### GA treatment decreases expression of CD11c

The fact that a soluble factor did not seem to be responsible for the early increase in phagocytosis led us to investigate the expression of several receptors involved in phagocytosis. We did not observe any differences in the expression of the integrin αM (CD11b), fatty acid translocase (CD36), integrin αVβ3 (CD51/61), low density lipoprotein receptor-related protein 1 (CD91), T cell immunoglobulin domain and mucin-like domain 3 (TIM-3), and macrophage mannose receptor 2 (CD206) after 24 h treatment with 31.25 µg/ml GA (Fig. 5C). For CD36, CD210, and TIM-3, analysis of surface expression was separately performed according to the monocyte subsets CD14^+^CD16^−^ and CD14^+^CD16^+^ cells (Table 4). However, there was no difference in the expression of these receptors as compared to the respective controls.

**Table 4:** Mean fluorescence intensities of CD36, CD210, and TIM-3 (± SEM) of three independent experiments after a treatment period of 24 h according to the different monocyte subsets, i.e. CD14^+^CD16^−^ and CD14^+^CD16^+^ monocytes.

Receptor	CD14^+^CD16^−^	CD14^+^CD16^+^
CD36 Medium	60.62 (±3.7)	141.37 (±1.9)
CD36 GA 31.25 µg/ml	55.83 (±16.3)	134.46 (±3.1)
CD210 Medium	154.34 (±13.7)	243.67 (±27.4)
CD210 GA 31.25 µg/ml	184.55 (±35.5)	262.39 (±25.9)
TIM-3 Medium	201.13 (±2.6)	99.83 (±28.2)
TIM-3 GA 31.25 µg/ml	210.28 (±10.2)	126.8 (±24.6)

Respective isotype controls did not differ from untreated controls without staining (data not shown).

**Figure 6:**
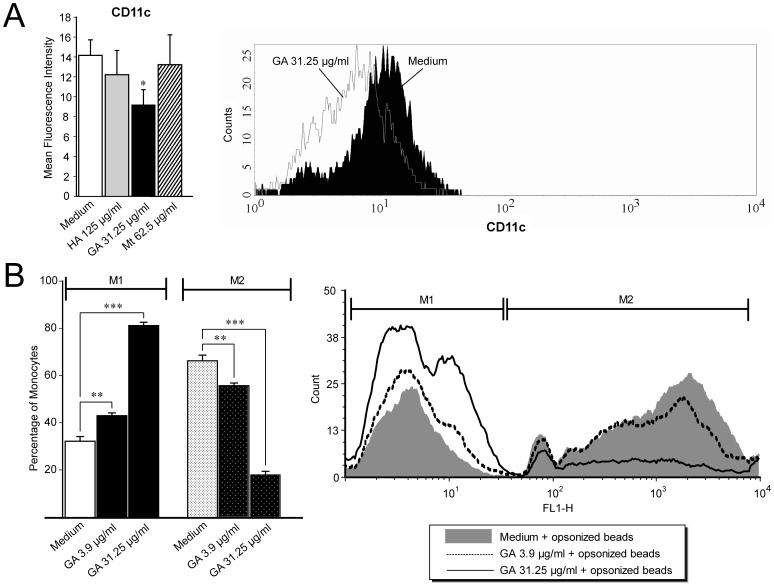
GA decreases CD11c expression. **A.** Significant decrease in CD11c expression after 24 h of GA treatment. **B.** 24 h treatment with 3.9 µg/ml and 31.25 µg/ml GA treatment dose-dependently decreased the percentage of phagocytosing monocytes (see M2 gate) and increased the percentage of non-phagocytosing monocytes (see M1 gate) of fibrinogen-coated beads. Data are expressed as mean percentages ± SEM of three independent experiments. Significant effects vs. GA 31.25 µg/ml or GA 3.9 µg/ml, respectively, are indicated by asterisks (*p<0.05, **p<0.01, and ***p<0.001 using Bonferroni's Multiple Comparison Test) as determined by one-way ANOVA.

CD11c (α-X subunit of the α-Xβ2 receptor), a member of the leukointegrin family, has been implicated in the phagocytosis of polystyrene beads and bacteria in the absence of complement [28]. Treatment with 31.25 µg/ml GA for 24 h significantly decreased the expression of this integrin (decrease of 35.1%) as compared to medium/isotype control (p = 0.012) (Fig. 6A). Of the different ligands that bind to CD11c, its binding to fibrinogen has been characterized most extensively [29]. GA treatment dose-dependently decreased the percentage of monocytes phagocytosing fibrinogen-coated polystyrene beads (4 µg/ml, 55.7%, SD±1.95, p = 0.01; 31.25 µg/ml, 17.9%, SD±2.48, p<0.001) and, in parallel, increased the percentage of non-phagocytosing cells (4 µg/ml, 43%, SD±2.03, p = 0.05; 31.25 µg/ml, 81.1%, SD±2.48, p<0.001) (Fig. 6B) as compared to medium control (phagocytosing cells, 66.2%, SD±4.1; non-phagocytosing, 32.19%, SD±3.36).

### Blockade of CD14, CD16, CD36, and CD210 reverses GA induced increase of phagocytosis

GA has been reported to interact in the unfolded state strongly with the integrin αMβ_2_ and inhibit myelin basic protein (MBP) binding to αMβ_2_ [17]. This study prompted us to assess the inhibitory effects of monoclonal antibodies raised against the integrins αM (Cd11b) and β_2_ (CD18). However, none of these antibodies interfered with the phagocytic activity. Although GA treatment diminished CD11c expression, an antibody directed to the α-X subunit of the α-Xβ2 receptor (CD11c/CD18) did not decline the phagocytosis either (data not shown). Furthermore, this antibody did not decrease the phagocytosis of beads coated with a CD11c ligand (fibrinogen) suggesting that it is not functional (data not shown).

**Figure 7:**
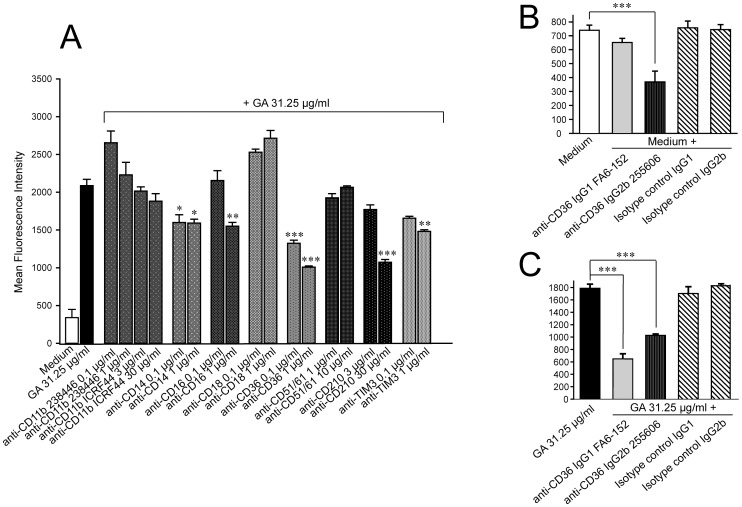
The phagocytosis of polystyrene beads was most significantly decreased by anti-CD36 antibodies. The MFI displays the amount of incorporated beads phagocytosed by 3×10^5^ cells. Data are expressed as means of the mean fluorescence intensities (MFI) ± SEM of three independent experiments. Pre-treatment of monocytes with anti-CD14, anti-CD16, anti-CD32, anti-CD210, and anti-TIM3 antibodies reduced glatiramer acetate (GA) induced phagocytosis of polystyrene beads. The anti-CD36 and, at the highest concentration, the anti-IL10 antibody most effectively suppressed phagocytosis. Significant effects vs. GA 31.25 µg/ml are indicated by asterisks (*p<0.05, **p<0.01, and ***p<0.001 using Bonferroni's Multiple Comparison Test) as determined by one-way ANOVA. **B–C.** The anti-CD36 antibodies of two different clones show a diverse impact on the phagocytosis of polystyrene beads by untreated cells, whereas both antibodies effectively decreased the GA induced phagocytosis.

In addition, the anti-CD14 antibody at both concentrations (0.1 and 1 µg/ml) reduced the bead uptake by approximately 23–24% (p = 0.026, p = 0.021), whereas the anti-CD16 antibody limited the uptake only at the highest concentration (1 µg/ml, decrease of 25.8%, p = 0.008) (Fig. 7A).

As mentioned above, treatment with IL-10 did not lead to an early increase of the bead phagocytosis as compared to medium control. However, the antibody targeting the IL-10 receptor (CD210) effectively diminished the phagocytic activity by approximately 49% (p<0.001) at 30 µg/ml (Fig. 7A). Finally, the anti-TIM3 antibody appeared to inhibit phagocytosis at the highest concentration (1 µg/ml, decrease of 29%, p = 0.002) suggesting a possible involvement in GA mediated phagocytosis.

**Figure 8:**
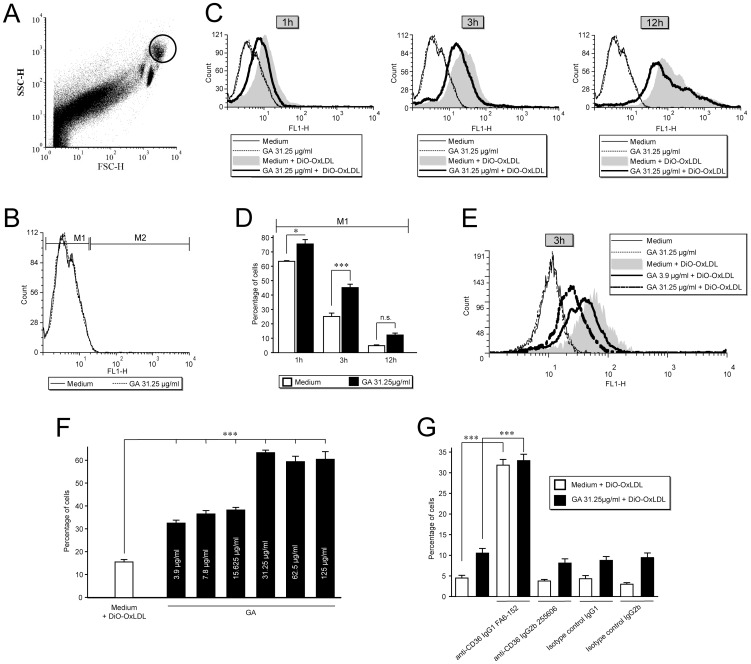
Anti-CD36 antibodies did not revert the GA mediated inhibition of OxLDL uptake. **A.** Monocytes were gated within PBMC according to their light scatter characteristics. **B.** Glatiramer acetate (GA) and medium control without any preceding treatment within the marker M1 served as a control for the analysis of monocytes that did not ingest labelled low density lipoprotein (DiO-OxLDL). **C.** At the indicated timepoints GA treated cells exhibited a slight left-shifted fluorescence signal, which was most prominent after 3 h of DiO-OxLDL incubation. **D.** Data are expressed as the percentages of monocytes that did not ingest DiO-OxLDL. After 1 h and 3 h the percentages of those cells were significantly higher in GA treated cells than in untreated cells. **E–F.** The percentages of non-ingesting cells are higher at higher concentrations as compared to lower concentrations of GA suggesting a dose-depending effect. **G.** The anti-CD36 antibody from the clone FA6–152 distinctly increased the amount of non-ingesting cells, while another anti-CD36 antibody (clone 255606) did not have an impact.

The integrin αVβ3 (CD51/CD61) has been proposed to act in concert with CD36 [30]. Intriguingly, the anti-CD36 antibody (clone 255606) most effectively suppressed the phagocytosis up to approximately 36 to 52% (p<0.001), but the antibody against the integrin αVβ3 (CD51/61) did not influence phagocytosis suggesting that in the engulfment of latex beads these receptors are not operating in parallel (Fig. 7A). A suppressive effect was also observed with another anti-CD36 antibody (clone FA6–152) that inhibited phagocytosis by 31 to 40% (p<0.001) at 1 µg/ml (Fig. 7C). While this antibody did not suppress the phagocytosis in untreated monocytes, the anti-CD36 antibody from the clone 255606 significantly reduced this “spontaneous” phagocytosis by 39 to 60% (p<0.001) at a concentration of 1 µg/ml (Fig. 7B). We additionally chose labelled Ox-LDL, known to be endocytosed into the cell via the CD36 receptor, in order to test whether these antibodies are specific in relation to the GA mediated effect. The percentage of cells that did not ingest Ox-LDL after 1 h (mean percentage of cells: 63.5%, SD±0.8) and 3 h (mean percentage of cells: 25.1%, SD±4.1) of incubation with 10 µg/ml DiO-OxLDL was significantly higher if they were pretreated with 31.25 µg/ml GA for 3 h (1 h, mean percentage of cells: 63.5%, SD±5.3, p = 0.014; 3 h, mean percentage of cells: 45.2%, SD±4.1, p<0.001) (Fig. 8C–D). However, no significant differences were seen after 12 h of incubation with 10 µg/ml DiO-OxLDL (GA vs. medium control, p = 0.296) (Fig. 8D). Moreover, an increase in the cell number not internalizing DiO-OxLDL after 6 h of incubation was even observed for monocytes pretreated with lower GA concentrations (Fig. 8E–F). While lower concentrations of GA (3.9–15.625 µg/ml) resulted in similar cell percentages, a significant increase in the percentage of cells which did not ingest DiO-OxLDL was noticed at a GA concentration of 31.25 µg/ml (GA 31.25 µg/ml vs. lower concentrations, p<0.001) suggesting a dose-dependent effect. However, a further increase of the GA concentration, up to 125 µg/ml, did not further increase the percentage of phagocytosing cells.

Both anti-CD36 antibodies showed a disparate impact on the endocytosis of DiO-OxLDL. The anti-CD36 antibody from the clone 255606 did not interfere with the amount of endocytosing cells, while the anti-CD36 antibody from the clone FA6–152 impressively increased the amount of non-endocytosing cells (Fig. 8G). However, we could not detect that both antibodies might have even partially reverted the effect of GA suggesting that their inhibition on the phagocytosis of polystyrene beads was unspecific and not related to the mode of GA action.

For all blocking experiments, monocytes were gated within PBMC according to the light scatter characteristics (Fig. 8A). Factors like a changed autofluorescence by GA (Fig. 8B) or cytotoxicity by the anti-CD36 antibodies were excluded (Table 5 and 6). None of the other antibodies (Fig. 7A) at the concentrations used induced a decrease in cell viability which may cause an artificial decline of bead engulfment (data not shown). Corresponding isotype controls with successive GA treatment did not influence phagocytosis as compared to the (31.25 µg/ml) GA control (data not shown).

**Table 5:** Percentages of monocytes positive for either annexin V, PI or both obtained from gate properties of the FL1 and FL3 sensor treated with an anti-CD36 antibody, clone 255606, in low (0.1 µg/ml) and high (1 µg/ml) concentrations, with or without 31.25 µg/ml Glatiramer Acetate (GA).

Clone 255606	Annexin V (positive)	Annexin V & PI (positive)	PI (positive)
Medium	10.69% (±4.4)	6.22% (±0.2)	0.27% (±0.1)
αCD36_low_	9.83% (±4.8)	5.10% (±0.2)	0.65% (±0.4)
αCD36_high_	10.05% (±3.7)	4.48% (±1.1)	0.45% (±0.3)
αCD36_low_+GA	9.39% (±3.8)	10.09% (±1.8)	0.77% (±0.2)
αCD36_high_+GA	8.39% (±3.3)	7.93% (±2.5)	0.54% (±0.3)

**Table 6:** Percentages of monocytes positive for either annexin V, PI or both obtained from gate properties of the FL1 and FL3 sensor treated with an anti-CD36 antibody, clone FA6–152, in low (0.1 µg/ml) and high (1 µg/ml) concentrations, with or without 31.25 µg/ml Glatiramer Acetate (GA).

Clone FA6–152			
Medium	8.93% (±3.4)	5.59% (±3.8)	0.39% (±0.3)
αCD36_low_	9.03% (±2.1)	6.04% (±3.6)	0.36% (±0.1)
αCD36_high_	9.96% (±3.3)	6.40% (±4.6)	0.52% (±0.3)
αCD36_low_+GA	8.09% (±5.1)	7.89% (±1.6)	0.30% (±0.1)
αCD36_high_+GA	8.19% (±2.2)	7.62% (±0.7)	0.43% (±0.4)

## Discussion

GA has been shown to alter the activation state and cytokine pattern of a variety of different APC resulting in anti-inflammatory effects on all cell types constituting the innate immune system. While Th2 skewing of the T cell response is a widely accepted mode of action, the induction of anti-inflammatory APCs, so-called “type II” APCs, is an emerging new concept for this substance. We recently reported that upon GA treatment rat microglia exerted a distinctly amplified phagocytosis of polystyrene beads [16]. However, as a mixture of four synthetic polypeptides (50–90 amino acids, average molecular weight 5–9 kDa) of high polarity and hydrophilic nature a direct effect of GA on the CNS seems rather implausible curtailing its “operating area” exclusively to the periphery.

A feasible target in the periphery are cells of the monocyte/macrophage lineage. Indeed, *ex vivo* isolated monocytes from GA treated MS patients have already been shown to respond with lower efficiency to stimulation with LPS, to secrete more IL-10 and less IL-12 [10], [14]. With the present work we are broadening this line of evidence by showing that GA treatment augments phagocytosis *in vivo* as demonstrated on *ex vivo* isolated monocytes. Our successive *in vitro* experiments delineate that this effect appears fast and does not result from autocrine stimulation. Even at low concentrations of GA (3.9 and 7.8 µg/ml) phagocytosis was reliably induced which could be of importance in case that after GA injection only a small amount of the injected material, either intact or partially hydrolyzed, may enter the lymphatic or systemic circulation.

Intriguingly, phagocytosis was confined to a small subset of monocytes which are positive for CD16 and exhibit a higher phagocytic activity especially in the CD14^++^CD16^+^ subset. According to a recent nomenclature proposal of monocytes, the CD14^++^CD16^+^ subset shall be referred to as intermediate monocytes [23]. Elevated counts of such monocytes have been reported in several pathologic conditions [31], [32], [33], [34], [35]. Although these monocytes were first described in 2003, they still represent a poorly characterized subset [36]. CD16-positive monocytes are in general considered to be “pro-inflammatory” cells as stimulation by the Toll-like receptor (TLR)-4 ligand LPS or the TLR-2 ligand Pam3Cys results in a higher expression of TNF-α and a lower expression of anti-inflammatory IL-10 [24]. Accordingly, after stimulation with LPS, the production of IL-1β and TNF-α has been shown to be restricted to intermediate monocytes. In contrast to non-classical monocytes (CD14^+^CD16^+^), intermediate monocytes are able to produce IL-10 as well [37], [38]. However, since the proportion of these cells amongst monocytes is less than 10%, it seems rather unlikely that they have a gross impact on the cytokine milieu in the blood circulation. Although we observed an increase of the proportion of CD16-positive monocytes up to approximately 24 percent *in vitro*, this may not transfer into a cytokine shift but could be an explanation for the increased phagocytic activity *in vivo*.

Intermediate monocytes are endowed with an array of proteins responsible for adhesion, migration, and phagocytosis [39]. For example, amongst the different monocyte subsets, the expression of CXCR1 has been shown to be the highest in CD14^++^CD16^+^ monocytes. Its ligand, fractalkine (CX_3_CL1), has been shown to mediate arrest and migration of CD16-positive monocytes suggesting that these cells preferentially recruit to endothelium, migrate into tissue, and phagocytose [36]. Several functional studies could confirm the highest phagocytic capacity in CD14^++^CD16^+^ monocytes which is in line with our data [37], [39]. Although it stands to reason that intermediate or non-classical monocytes may cross the blood brain barrier, their specific roles in the CNS, particularly in the context of MS, remain absolutely unclear. The phagocytic potency of intermediate monocytes, for example, may be of relevance for removal of myelin debris which is a crucial step for remyelination [40], [41], [42], [43].

Recombinant interferon-β (IFN-β), like GA a basic disease-modifying treatment for MS, has been reported to induce in MS patients a phenotypic shift from CD14^+^CD16^+^ towards CD14^++^CD16^+^ monocytes which simply means that the CD14 expression is increased by IFN-β [44]. In contrast, we observed that GA treatment resulted in a phenotypic shift from CD14^++^CD16^−^ towards CD14^++^CD16^+^ cells *in vitro*, whilst the CD14 expression was not altered. Although not attaining statistical significance, this was accompanied by a trend towards increased CD16 expression. It has to be mentioned that in our *in vitro* setting monocytes already exhibited a high CD14 expression resulting in either CD14^++^CD16^−^ or in CD14^++^CD16^+^ monocytes. Due to this experimental bias, an additional increase of the CD14 expression thus cannot be excluded.

It was suggested by Stapulionis et al. that GA interacted in the unfolded state strongly with CD11b [17]. In addition to this work, the intravenous injection of Alexa Fluor® 488-labelled GA has recently been reported to result in a specific binding of GA to CD11b^+^ F4/80^lo^ Ly6G^−^ blood monocytes of C57BL/6J mice via an MHC class II–independent mechanism [45]. Of major concern is the fact that GA applied as a whole protein mix will be most probably not specific for a single receptor, if at all. Nevertheless, these two observations prompted us to examine the expression of several phagocytic receptors including CD11b. Apart from CD11c, we could not detect any significant changes in the expression of these receptors as compared to controls. As mentioned above, CD11c has been proposed to be involved in the uptake of non-opsonized polystyrene beads [28]. Another approach by performing the phagocytosis assay with beads coated with a CD11c ligand substantiated the decrease in the CD11c receptor in a functional manner. Nevertheless, the change in CD11c expression may result from a GA induced differentiation of monocytes possibly being unrelated to phagocytosis. These results are very interesting in view of the fact that studies with Chinese hamster ovary (CHO) fibroblasts have shown that transfection of CD11c/CD18 rendered the cells responsive to LPS and Gram-negative bacteria, independently of CD14 [46]. The decrease in CD11c expression might explain the reduced reactivity of GA treated monocytes in response to TLR-4 stimulation [14].

In our blocking assays, treatment with anti-CD14, anti-CD16, anti-CD36, anti-CD210, and anti-TIM3 antibodies led to a decrease in phagocytosis suggesting that more than one surface protein/receptor is involved in bead engulfment. In particular, treatment with anti-CD36 antibody clearly halved the phagocytic activity. The class B scavenger receptor CD36 is implicated in a variety of cellular processes including lipid uptake, inflammation, and as a pattern recognition receptor mediating innate immune responses [47]. We suspected that this receptor could be responsible for the GA induced phagocytosis and, thus, focused on performing further experiments with an additional anti-CD36 antibody from a different clone. The decrease in bead phagocytosis of untreated monocytes by one of these antibodies already indicated that blocking of the CD36 receptor is very likely not linked to GA. In addition, we assessed the ingestion of labelled human oxidized low density lipoprotein (Ox-LDL), which is well-known to be a ligand of CD36 [48]. GA significantly inhibited the uptake of this lipoprotein as constrained by an increase of the percentage of non-ingesting cells. However, both antibodies failed to revert even partially the effect of GA in this context. Although CD36 was a quite promising candidate, GA does not seem to bind to this receptor. Interestingly, the inhibitory effect of GA on Ox-LDL was surprising and as yet not described. Mosig et al. reported that monocytes with a high phagocytosis rate took up only small amounts of Ox-LDL and, vice versa, monocytes with a high uptake of Ox-LDL had a reduced ability for the ingestion of polystyrene beads [49]. Thus, we suspect that the inhibitory effect of GA on Ox-LDL may be due to a functional change of monocytes.

The decrease in the expression of the CD11c receptor and the increase in the percentage of monocytes of the intermediate subset warrant further *in vivo* confirmation. Since there is currently no information about the absorption, distribution, metabolism or excretion profile of GA in humans, which would enable the use of physiological concentrations, we have to note that the GA concentrations used in our *in vitro* experiments are similar to those used in many other *in vitro* studies [13], [14], [15], [50].

In summary, our results demonstrate that GA treatment augments phagocytic activity of monocytes *in vivo* and thus adds a new mechanism of action of GA in MS patients. Our *in vitro* experiments delineate that this activity arises from the intermediate CD14^++^/CD16^+^ monocyte subset. Moreover, GA significantly decreases the expression of the CD11c receptor and dose-dependently blocks the phagocytosis of beads coated with a CD11c ligand.
